# The paradox of local inequality: Meritocratic beliefs in unequal localities

**DOI:** 10.1111/1468-4446.12930

**Published:** 2022-03-08

**Authors:** Katy Morris, Felix Bühlmann, Nicolas Sommet, Leen Vandecasteele

**Affiliations:** ^1^ LIVES Centre, Swiss Centre of Expertise in Life Course Research Lausanne Switzerland

**Keywords:** England, local inequality, meritocracy, meritocratic beliefs | income inequality

## Abstract

A puzzle has emerged amidst rising inequality: why do people profess high levels of belief in meritocracy even as income gains are increasingly concentrated at the top? In light of contradictory theories and evidence, we undertake the first assessment of the relationship between local income inequality and meritocratic beliefs outside the United States, using data from the UK Household Longitudinal Study. We find that the positive relationship between country‐level income inequality and meritocratic beliefs identified in the recent literature does not translate straightforwardly below country level: there is no robust relationship between local income inequality and meritocratic beliefs in England. However, there is a robust—and somewhat paradoxical—positive association between high local income inequality and meritocratic beliefs among those with the lowest incomes. On average, respondents with annual household incomes of £10,000 are five points more likely (on a 100‐point scale) to believe their hard work will pay off if they live in the most rather than the least unequal places in England. We also show that this applies beyond the specific case of meritocratic beliefs: low‐income respondents in unequal places are also notably more satisfied with their own (low) income than similar respondents in more equal localities. In line with system justification theory, we argue that belief in meritocracy serves as an important tool of psychological resilience for low‐income individuals who regularly come into contact with others more economically fortunate than themselves: though it legitimates their current position at the bottom of the status hierarchy, this belief also offers the promise of future advancement. While this reduces concern about the psychological effects of growing local income inequality on the most economically vulnerable, it also suggests that there is little prospect of demand for systemic economic change emerging from what might have been considered the most likely places.

## 1 INTRODUCTION

The concept of meritocracy has walked a winding historical path. Invented as a dystopian satire (Young, 1958), the idea that social status and financial success follow on from individual talent and effort and that people advance on the basis of their merits, has evolved into one of the central underpinnings of Western democracies. In recent times, it has not only become the source of substantial criticism, on the basis that enables the dominant to legitimize their superior position (Bloodworth, 2016; Friedman & Laurison, 2019; Littler, 2017; Markovits, 2020; McNamee & Miller, 2004; Sandel, 2020) but also the source of considerable puzzlement. Why is it that public belief in meritocracy has held steady or indeed increased over time, even as income inequality has grown?

The peculiar relationship between country‐level income inequality and meritocratic beliefs is documented by Mijs (2020), who shows that the citizens of countries with higher levels of income inequality—and, per the Great Gatsby curve, lower levels of social mobility (Durlauf & Seshadri, 2018)—are more likely to attribute success to meritocratic factors than the citizens of more equal countries. Mijs (2020) and Mijs and Savage (2020) explain this “paradox of inequality” with reference to higher levels of social and spatial distance in more unequal countries. They argue that higher income inequality reduces opportunities for mixing and interaction across income lines. As a consequence, they maintain, both rich and poor increasingly resort to meritocratic rather than structural explanations of their own situations.

This explanation raises important questions about the relationship between local contexts and meritocratic beliefs. Though a simple extrapolation of Mijs' (2020) findings might suggest that people who live in more unequal localities would hold stronger meritocratic beliefs, his emphasis on the importance of interactions across economic fault lines instead indicates the opposite. Unequal localities are by definition places where residents are more likely to regularly encounter diversity and social otherness, and where it is consequently harder to maintain the perception of deservingness. On this basis, it may well be the case that people in more unequal localities hold weaker meritocratic beliefs.

Existing evidence on this topic is contradictory. Exploring the relationship between local income inequality and meritocratic beliefs in the United States, both Newman et al. (2015) and Solt et al. (2016) identify important differences in the effect of local income inequality between low and high earners, but in opposite directions. Consistent with the idea that residing in a highly unequal locality tends to increase individual awareness of relative economic status (Festinger, 1954; Runciman, 1966; Wilkinson, 1997), Newman et al. (2015) find that higher levels of local income inequality are associated with polarised belief in meritocracy across income lines: low‐income individuals are more likely to reject and high‐income individuals more likely to endorse meritocratic ideology if they live in a highly unequal locality. Undertaking a similar analysis but with a larger sample of American respondents, Solt et al. (2016) conclude that the opposite is in fact true: low‐income respondents tend to express a stronger belief in the meritocracy if they live in high inequality localities. This, they maintain, offers further evidence of the validity of relative power theory, which holds that higher levels of inequality enable wealthier citizens to reshape the political landscape to their own advantage.

While Newman et al. have subsequently acknowledged two sets of errors^1^ in their original analysis, these authors maintain that “the core results of the published article remain unchanged” (2016a, p. 806) when the initial errors are corrected. To test the Mijs' (2020) spatial distance hypothesis and help resolve the Newman‐Solt controversy, we therefore undertake the first assessment of the relationship between local income inequality and meritocratic beliefs outside the United States, using data from the UK Household Longitudinal Survey (UKHLS). In doing so, we make three contributions to a broader understanding of this important issue. First, we show that the positive relationship between country‐level income inequality and meritocratic beliefs does not translate straightforwardly below country level: there is no robust relationship between local income inequality and the meritocratic beliefs of just under 25,000 UKHLS respondents in England. But second, and in line with Solt et al. (2016), we show that there is a small but robust—and somewhat paradoxical—positive association between local income inequality and meritocratic beliefs among low‐income respondents in England. Third, we challenge the relative power theory explanation offered by Solt et al. (2016), since this theory cannot easily explain why low‐income respondents living in unequal places are also notably more satisfied with their own (low) income than similar respondents in more equal localities. Instead, we propose a system justification theory explanation, whereby belief in meritocracy serves as an important tool of psychological resilience for low‐income respondents who regularly come into contact with others more economically fortunate than themselves (Jost, 2020; Jost et al., 2004; Jost & Banaji, 1994; McCoy et al., 2013). While meritocratic beliefs legitimate their current position at the bottom of the status hierarchy, they also hold out the promise of future advancement.

The nature of the relationship between income inequality and meritocratic beliefs is fundamental to the broader questions of how (and how much) income inequality affects individual well‐being (Pickett & Wilkinson, 2010; Wilkinson & Pickett, 2006) and whether such inequality is ultimately self‐correcting, via the ballot box (Kelly & Enns, 2010; Scheidel, 2017; Solt et al., 2017). In individual terms, our findings highlight the psychological resilience of the most economically vulnerable members of society in the face of high levels of local income inequality. The paradox of local inequality is that the same resilience potentially contributes to the justification and maintenance of the very order that produces these economic vulnerabilities in the first place.

## 2 STATE OF THE LITERATURE

### 2.1 The paradox of inequality

Mijs (2020) coins the term “the paradox of inequality” to describe the puzzling trend by which belief in meritocracy tends to be higher in countries with higher levels of income inequality. He compares the relationship between income inequality and beliefs in individuals (i.e., meritocratic) and structural inequality, both between countries and within countries over time. All else being equal, the citizens of the most unequal of the 43 country periods in his sample report meritocratic beliefs that are approximately 12 points higher (on a 100‐point scale) than those of the most equal countries.

In the first part of our analysis, we seek to test the explanation of these findings that Mijs (2020) offers, which can be thought of as a two‐pronged form of the contact hypothesis (Allport, 1957). First, Mijs (2020, p. 6) states, *what grounds people's beliefs about inequality is their exposure to and interactions with other people across economic fault lines*. In other words, direct contact with, and indirect exposure to, the economic “other” illuminates the structural forces that create and shape material inequalities, while interactions within homogenous economic contexts obfuscate these structural forces and lead people to seek individual explanations of inequality, chief among them meritocracy. Second, he argues that the growth of income inequality has reduced opportunities for interaction (in the firm of direct contact or indirect exposure to signs of poverty or affluence) across economic fault lines, with rich and poor increasingly living their lives in separate spheres. The growth of meritocratic ideology reflects the increasing social and spatial distance between income groups and the fact that, *as the gap grows larger, other people's lives fade out of view* (Mijs, 2020, p. 6).

This emphasis on proximity to the economic other points to the existence of a negative relationship between local income inequality and meritocratic beliefs that may at first seem counterintuitive, given the positive nature of this relationship at the country level. The expectation of a negative relationship reflects the fact that highly unequal localities are, by statistical definition, places that contain more of an economic mix than equal localities. They are consequently places in which residents are more likely to regularly encounter the economic diversity and social otherness that Mijs (2020) argues that it is fundamental to the development of a more structural and less individualistic understanding of inequality.^2^ If so, then we expect that people in more unequal localities will hold weaker meritocratic beliefs than those in more equal places. Our first hypothesis (H1) is therefore: *the more unequal a local context, the weaker meritocratic beliefs will be*.

### 2.2 Unequal localities: Activated conflict or relative power?

The relationship between local income inequality and meritocracy was already at the center of two prior studies by Newman et al. (2015) and Solt et al. (2016) in the United States. These two sets of authors offer a more nuanced picture, as the effect of local income inequality seems to depend on the position of individuals within the income distribution. However, much remains unclear because these authors advance contradictory theories and reach directly opposing conclusions about the nature and direction of this conditional relationship. The second part of our analysis therefore seeks to resolve this debate by exploring the relationship between individual income, local income inequality, and meritocratic beliefs outside the US context.

#### 2.2.1 Activated disillusionment and activated loyalty

In common with Mijs (2020), Newman et al. (2015) assume that inequality is experienced most directly on the local level and that local interactions across economic fault lines trigger particular reactions. But whereas the implication from Mijs (2020) is that such interaction will have the same effect on meritocratic beliefs irrespective of individual income, Newman et al. (2015) advance a theory of activated class conflict, which posits differential effects based on individual position within the income distribution.

Activated class conflict theory, which builds on earlier theories of social comparison (Festinger, 1954) and relative deprivation (Runciman, 1966), centers on the idea that *residence in high‐inequality contexts, compared to relatively equal contexts, increases the salience of economic comparisons and one's own relative economic position, and thus polarizes public belief in meritocratic ideology across income‐based lines* (Newman et al., 2015, p. 327). This is a two‐step process: daily confrontation with the economic other in unequal localities induces social comparison and makes everyone more aware of their social status (Alderson & Katz‐Gerro, 2016; Cheung & Lucas, 2016; Präg et al., 2014; Walasek & Brown, 2016). When activated, this “situational trigger” then reinforces “latent opinions” about inequality and transforms them into more explicit rejection or acceptance of meritocracy, depending on whether the individuals in question are at the bottom or the top of the income distribution. According to the activated disillusionment hypothesis, those *at the bottom of a more conspicuous local economic totem pole* (Newman et al., 2015, p. 329) are more likely to reject meritocracy as a means of protecting their self‐esteem (Cast & Burke, 2002). Conversely, the “activated loyalty hypothesis” holds that higher‐income Americans will be more likely to uphold meritocratic ideology if they live in more unequal contexts. Consistent with evidence that higher status individuals are more likely to see the existing social system as legitimate (Brandt, 2013; Brandt et al., 2020), Newman et al. (2015) argue that greater exposure to inequality increases high earners' awareness of their own privileged position and triggers both system and self‐justification processes.

Newman et al. (2015) find considerable support for their activated class conflict theory of meritocratic beliefs among white respondents^3^ in four nationally representative Pew surveys conducted between 2005 and 2009. For a sample of 6436 respondents, they find that those with household incomes below $10,000 and above $100,000 are, respectively, 8% points more likely and 6% points less likely to reject meritocracy if they live in the most rather than least unequal US counties. Our second subhypothesis (H2a) refers to this interaction between individual income and the local context: *the more unequal the local context in which low income people reside, the weaker their belief in meritocracy; for high income people, this association is attenuated*.

#### 2.2.2 Theories of relative power

However, the claims of Newman et al. (2015) have not gone uncontested. Having tried and failed to replicate their findings, Solt et al. (2016) challenge these authors' construction of the dependent variable and their interpretation of the key interaction term. Solt et al. (2016) then undertake a similar analysis with a much larger sample (*N* = 35,556) drawn from the US Religious Landscape Survey (RLS) and reach largely opposite conclusions. They find that low‐income respondents living in the most unequal counties are 19% points (±7) *less* likely to reject meritocracy than similarly low‐income people living where inequality is at its lowest observed level. Or formulated differently, low‐income respondents tend to express a stronger belief in the meritocracy if they live in high inequality localities. The same shift in local income inequality is associated with a decline in the predicted probability of rejecting meritocracy among individuals with household incomes below $50,000, but there is no relationship above this income threshold. We therefore also test the following counter subhypothesis (H2b): *the more unequal the local context in which low income people live, the stronger their belief in meritocracy; for high income people, this association is attenuated*.

Solt et al. (2016) interpret their results not only as a clear refutation of activated class conflict theory but also as further proof of the validity of relative power theory (Gaventa, 1980; Goodin & Dryzek, 1980; Kelly & Enns, 2010; Ritter & Solt, 2019; Solt, 2008, 2015; Solt et al., 2011). The central idea of relative power theory is that money is a power resource: where income and wealth are more concentrated, so too is the relative power of the rich. In highly unequal localities, Solt et al. (2016) contend that the wealthy are better able to spread self and system justifying values such as meritocracy to less affluent fellow residents, who lack the resources to resist these influences and are consequently more likely to internalize beliefs that justify the status quo. Solt et al. (2016) also complement their explanation with a psychological element: they argue that belief in meritocracy serves as an important source of resilience for the less well‐off because it provides *a means of escaping the subjective sense of powerlessness, if not its objective condition* (Gaventa, 1980, p. 17).

#### 2.2.3 Remaining questions

While all three accounts agree on the importance of local income inequality for meritocratic belief formation, they disagree not only on the expected direction of this relationship but also on the mechanisms involved. For Mijs and Newman et al. (2015), the level of inequality is important because it shapes the likelihood of contact and interactions across economic fault lines, which are assumed to trigger particular reactions. For Solt et al. (2016), local income inequality is important not because it affects the likelihood of encountering the economic other, but because it affects the degree of power wielded by the wealthy and the ability of the less affluent to resist these influences. By exploring the relationship between local income inequality and belief in the meritocratic ideal in a new country context, we aim to shed light on both the direction of and the mechanisms behind this relationship.

## 3 DATA AND METHOD

### 3.1 Data

Individual‐level data are drawn from Wave 5 of the UK Household Longitudinal Study (University of Essex, 2020a, 2020b, 2020c) which was undertaken over the period 2013–2015. The UKHLS is a longitudinal household survey that has followed individuals aged 16 and above within a nationally representative sample of approximately 40,000 households since 2009. This exemplary annual survey collects a wealth of information across a host of demographic, socioeconomic, and attitudinal domains, as well as data on the residential location of respondents and households within the United Kingdom.^4^

**FIGURE A1:**
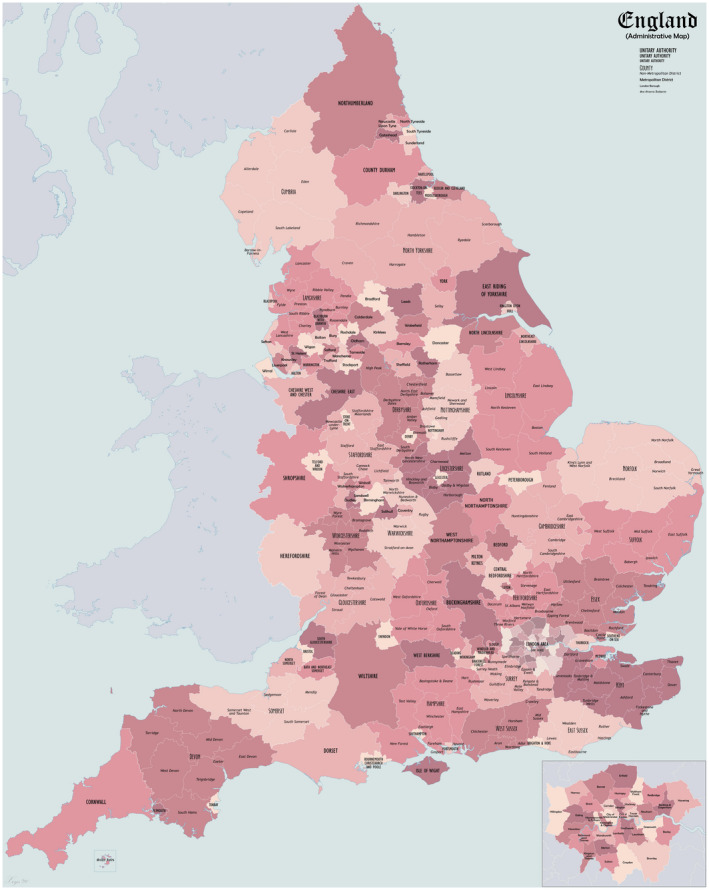
The 317 English Local Authority Districts (2019 boundaries). *Source*: Wikipedia user XrysD, https://commons.wikimedia.org/w/index.php?curid=10131879

Here, residential location refers to the 317 (2019 boundaries) English unitary authorities, metropolitan boroughs, nonmetropolitan districts, and London boroughs (Local Authority Districts or LAD hereafter) illustrated in Figure A1 in the Appendix. Local Authority Districts are autonomous local government units that are responsible for the provision of a range of facilities and services for the resident population. While LADs vary considerably in area and population size, they closely approximate the US counties used in the analysis by Newman et al. (2015) and Solt et al. (2016) and best capture the scale at which the interactions and exchanges which shape beliefs can be expected to take place. In England, the next available administrative unit below Local Authority Districts would be the 6791 Middle‐layer Super Output Areas (MSOAs),^5^ but research shows that people typically work, shop, socialize, and send their children to school across a much wider geographical area. That only 11% of UK employees work in the same MSOA that they live in (Fraja et al., 2020) and the average worker commutes 10 miles to work (Department for Transport, 2017) means the great majority of people are exposed to multiple alternative settings beyond the one they live in, at a scale well encapsulated by Local Authority Districts.

We match individual‐level location data to administrative data drawn from a range of sources, including the UK Labour Force Survey, Rae and Nyanzu's (2019a, 2019b) *English Atlas of Inequality*, and experimental admin‐based household income statistics produced by the Office for National Statistics (ONS) for the tax year 2015–2016. Due to LAD‐level data availability, we restrict our analysis to UKHLS respondents based in England. Following this restriction and listwise deletion, the analytical sample comprises 24,943 respondents in 315 Local Authority Districts in England.

### 3.2 Dependent variable

Our dependent variable is derived from a one‐off question asked in Wave 5 of the UKHLS, when respondents were asked to express their agreement or disagreement with the following statement: “I have always felt like my hard work would pay off in the end.” This question differs from the more general survey items employed as measures of meritocratic beliefs by Mijs (2020), Newman et al. (2015), and Solt et al. (2016) in two respects: in its individual (“I”) rather than general (“most people”) orientation, and in the timeframe in which returns to hard work are expected. However, it ultimately captures the same underlying concept: that hard work is (or can be expected to be) rewarded.^7^ For ease of interpretation, we follow Larsen (2016) and Mijs (2020) in multiplying responses on the original 0–10 Likert scale by 10 to construct a dependent variable that resembles a percentage, ranging from 0 (strongly disagree) to 100 (strongly agree). In line with previous research in the UK context (Duffy et al., 2021; Snee & Devine, 2018), Figure 1 shows that meritocratic beliefs are notably high across the sample: the mean response is 71.5 and the most common response is 100 (strongly agree).

**FIGURE 1:**
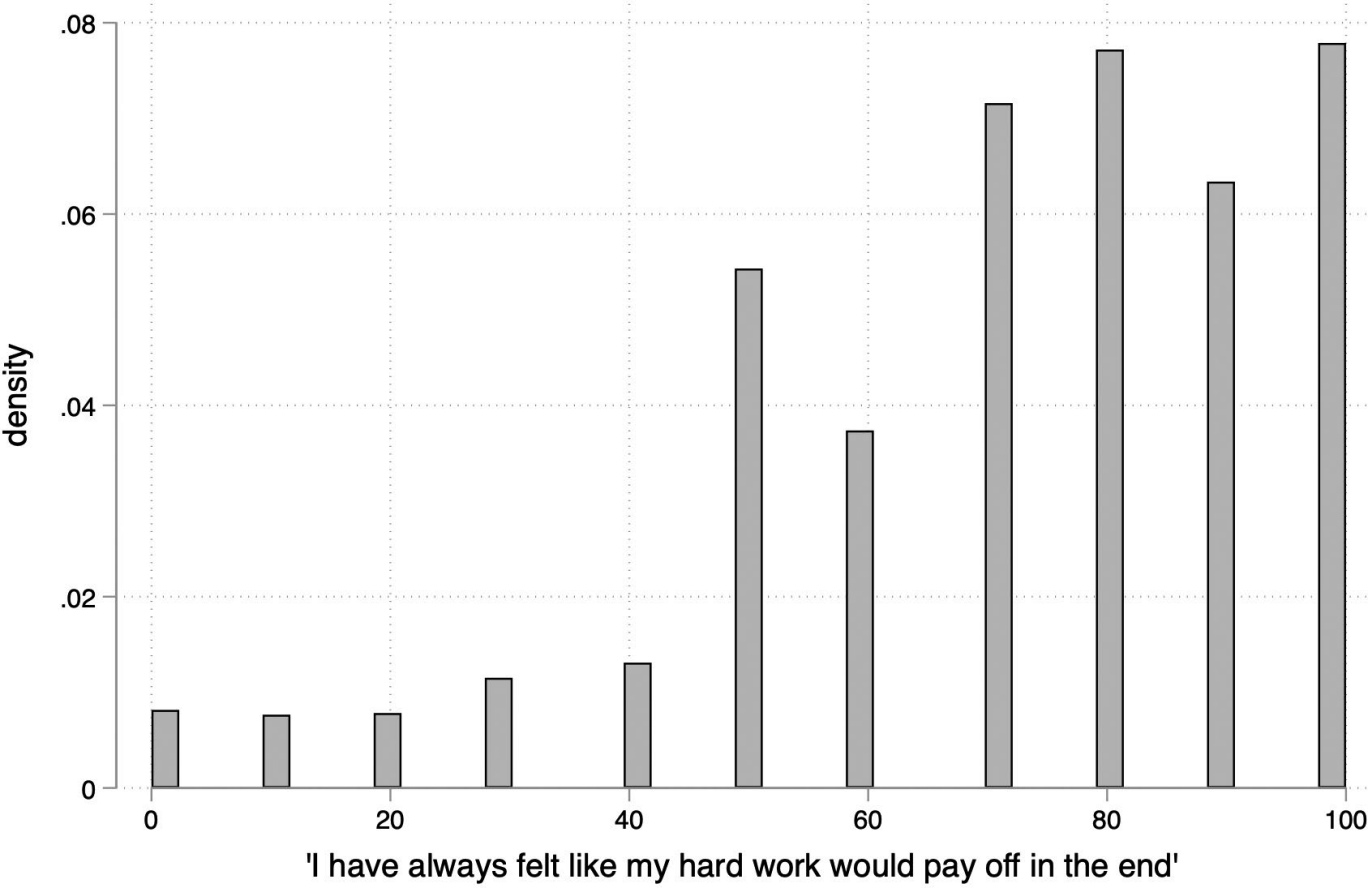
Meritocratic beliefs among UKHLS sample (*N* = 24,943), where 100 = strongly agree. *Source*: UKHLS Wave 5; graphical scheme by Bischof (2017)

### 3.3 Independent variables

In line with the great majority of research in this field, we measure income inequality at the Local Authority District level using the Gini coefficient of income inequality. The Gini coefficient describes the distribution of income within a particular spatial unit. It ranges from a minimum of 0 to a maximum of 1, whereby 0 would indicate that income is equally shared among all residents of an area and 1 that all income is held by 1 person or household. The United Kingdom is unusual in that the nine Government Office Regions represent the lowest spatial scale for which official ONS Gini data are available. We therefore use unofficial LAD Gini estimates constructed by Rae and Nyanzu (2019a, 2019b)^8^ using ONS data on household income from Pay As You Earn (PAYE) and welfare benefits for the tax year 2015–2016.^9^ At LAD level, the Gini coefficient ranges from 0.29 in Boston to 0.43 in Kensington and Chelsea, against a LAD‐level average of 0.34.

**FIGURE A2:**
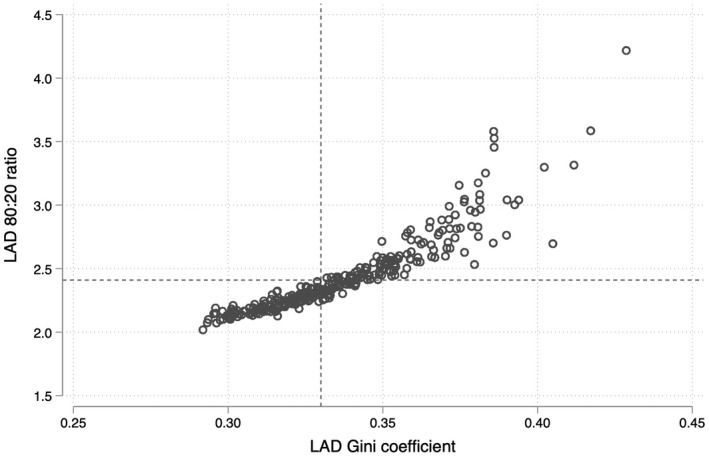
Comparing Local Authority District income inequality metrics

Though evidence suggests that residents are able to detect higher and lower levels of income inequality as measured by the Gini coefficient (Newman et al., 2018), it is also the case that Gini coefficients can be skewed by the presence of a very small number of extremely wealthy individuals, whose presence may not be obvious to the local population as a whole. To test the robustness of our findings, we therefore also compute and employ an alternative 80:20 measure of local income inequality, using ONS experimental admin‐based income statistics covering the tax year 2015–2016.^10^ The 80:20 metric is a simple ratio of the 80th percentile of net household income (in GBP) within each Local Authority District divided by the 20th percentile, with larger values indicating greater income inequality. Among Local Authority Districts, the 80:20 ratio ranges from 2.02 in Boston to 4.22 in Kensington and Chelsea, against a country‐level average of 2.41, and correlates with the Gini coefficient at 0.77 (see Figure A2 in the Appendix).

We follow Newman et al. (2015) and Solt et al. (2016) in incorporating three control variables at the LAD level in order to account for the effects of variation in demographic and socioeconomic conditions across localities. These are the total population of each LAD drawn from ONS Mid‐Year Population Estimates (2015); the proportion of residents from ethnic minority backgrounds derived from the 2015 Labour Force Survey; and median net household income in GBP, which is sourced from the same set of experimental ONS income statistics as the 80:20 ratio.

At the individual level, our main independent variable of interest is net annual household income (in thousands) in GBP. Reflecting the approach adopted by Newman et al. (2015) and Solt et al. (2016), we also control for gender, age and age squared, ethnicity, educational attainment, unemployment status, whether respondents belong to a religion, and their political leanings (right, left, other). Since any effect of local income inequality might reasonably be expected to be stronger among longstanding residents who have had more time to notice their surroundings than new arrivals, we additionally control for whether respondents have lived in the same Local Authority District since entering the survey. Descriptive statistics and coding schemas for all LAD and individual‐level variables are displayed in Table 1.

**TABLE 1:** Descriptive statistics for UKHLS Wave 5 sample

Variable	Coding	Min	Max	Mean	SD
Meritocratic belief		0	100	71.49	23.87
LAD population (000s)		38	1113	246.75	181.12
LAD % ethnic minority population		0.00	64.90	12.96	15.09
LAD median net household income (£ 000s)		19	32	24.02	2.32
LAD Gini coefficient		0.29	0.43	0.34	0.02
LAD 80:20 ratio		2.02	4.22	2.41	0.26
Net household income (£ 000s)		0	1699	37.55	36.51
Sex	0 = female 1 = male	0	1	0.55 0.45	
Age		16	101	48.35	17.84
Age squared		256	10201	2656.22	1794.65
Ethnicity	0 = white 1 = ethnic minority	0	1	0.20 0.80	
Education	0 = non‐graduate 1 = graduate	0	1	0.62 0.38	
Employment status	0 = employed or other 1 = unemployed	0	1	0.96 0.04	
Political leanings	1 = left 2 = right 3 = other	1	3	0.43 0.33 0.24	
Religious affiliation	0 = no affiliation 1 = belongs to a religion	0	1	0.50 0.50	
Lives in the same LAD as when entered UKHLS	0 = different LADs 1 = same LAD	0	1	0.10 0.90	

### 3.4 Method and models

We use linear multilevel models (estimated using the maximum likelihood estimator) to account for the hierarchical structure of our data, whereby individuals are nested in Local Authority Districts. In the first step, we estimate models with all controls in order to establish whether and how much LAD‐level income inequality affects meritocratic beliefs, all else being equal. In a second step, we introduce a cross‐level interaction between net household income and the LAD Gini coefficient (or 80:20 ratio) to test the hypothesis that the effect of income inequality is conditional on household income and establish the nature and direction of this relationship. All interaction models include a random slope on net household income as advised by Heisig and Schaeffer (2019), and all models are weighted using the cross‐sectional Wave 5 weights provided by the UKHLS.

## 4 RESULTS

### 4.1 Main analysis: Gini coefficient

Model I in Table 2 displays the results of our Step 1 regression model, which do not conform to the expectations of Hypothesis 1. The Gini coefficient has a small positive effect on meritocratic beliefs, but the estimate is imprecise. We therefore cannot reject the null hypothesis that local income inequality has no effect on belief in meritocracy among our sample as a whole.

**TABLE 2:** Linear mixed models of meritocratic beliefs among UKHLS respondents

	LAD GINI		LAD 80:20 RATIO	
	(I)	(II)	(III)	(IV)
Fixed effects				
LAD level				
LAD population (000s)	0.001	0.001	0.001	0.001
	(−0.001 – 0.003)	(−0.001 – 0.004)	(−0.001 – 0.003)	(−0.001 – 0.004)
LAD % BAME	−0.031	−0.030	−0.033*	−0.031
	(−0.071 – 0.008)	(−0.071 – 0.011)	(−0.073 – 0.006)	(−0.072 – 0.010)
LAD median income (£000s)	−0.153	−0.182*	−0.194*	−0.223*
	(−0.349 – 0.042)	(−0.382 – 0.019)	(−0.425 – 0.036)	(−0.457 – 0.010)
LAD Gini	11.798	45.967***		
	(−9.010 – 32.607)	(18.273 – 73.661)		
LAD 80:20 ratio			1.397	3.261***
			(−0.651 – 3.445)	(1.044 – 5.479)
Cross‐level interaction				
LAD Gini * income		−0.943***		
		(−1.472 – −0.414)		
LAD 80:20 ratio * income				−0.049***
				(−0.081 – −0.016)
Individual level				
Net household income (£000s)	0.021**	0.379***	0.021**	0.176***
	(0.001 – 0.042)	(0.195 – 0.563)	(0.001 – 0.042)	(0.089 – 0.262)
Male	−0.428	−0.505	−0.428	−0.500
	(−1.137 – 0.280)	(−1.207 – 0.197)	(−1.137 – 0.280)	(−1.201 – 0.202)
Age	−0.196***	−0.206***	−0.196***	−0.206***
	(−0.304 – −0.088)	(−0.314 – −0.098)	(−0.304 – −0.088)	(−0.314 – −0.098)
Age squared	0.002***	0.003***	0.002***	0.003***
	(0.001 – 0.003)	(0.002 – 0.004)	(0.001 – 0.003)	(0.002 – 0.004)
BAME	3.002***	3.071***	2.996***	3.058***
	(1.934 – 4.071)	(1.995 – 4.146)	(1.930 – 4.061)	(1.988 – 4.128)
Graduate	2.715***	2.364***	2.716***	2.379***
	(1.980 – 3.450)	(1.654 – 3.073)	(1.982 – 3.449)	(1.668 – 3.091)
Unemployed	−4.828***	−4.326***	−4.841***	−4.388***
	(−6.706 – −2.951)	(−6.187 – −2.465)	(−6.721 – −2.962)	(−6.256 – −2.521)
Belongs to a religion	1.904***	1.839***	1.901***	1.836***
	(1.140 − 2.668)	(1.082 − 2.596)	(1.138 − 2.665)	(1.080 − 2.593)
Political leanings	2.546***	2.395***	2.544***	2.390***
	(1.721 − 3.371)	(1.581 − 3.210)	(1.719 − 3.370)	(1.574 − 3.206)
	−0.578	−0.537	−0.582	−0.552
	(−1.493 – 0.337)	(−1.455 – 0.381)	(−1.497 – 0.334)	(−1.471 – 0.368)
Lives in the same LAD	−1.561***	−1.719***	−1.566***	−1.716***
	(−2.626 – −0.496)	(−2.789 – −0.650)	(−2.632 – −0.500)	(−2.786 – −0.647)
Constant	72.012***	60.020***	73.643***	68.760***
Random effects				
Income		0.001		0.001
LAD (constant)	1.380	0.751	1.379	0.596
Residual	554.962	552.264	554.946	552.650
Number of respondents	**24,943**	**24,943**	**24,943**	**24,943**
Number of LADs	**315**	**315**	**315**	**315**

*Source*: UKHLS Wave 5. Confidence intervals in parenthesis.

*** *p* < .001

** *p* < .01

* *p* < .05.

Bold value indicates sample N

**FIGURE 2:**
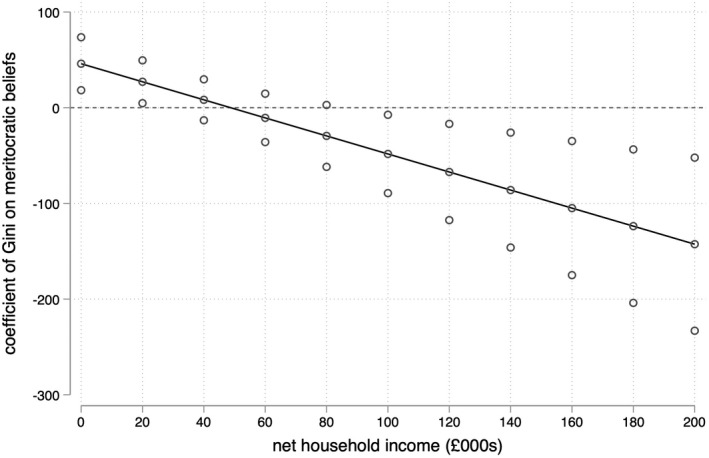
Coefficient of local income inequality on meritocratic beliefs by household income

Model II in Table 2 displays the results of our Step 2 regression model, which yields support for Hypothesis 2b and against Hypothesis 2a in the English context: higher levels of inequality are associated with stronger meritocratic beliefs among low‐income respondents. This can be seen in Figure 2, which plots the conditional effect of local income inequality at different observed values of household income. Figure 2 shows that the estimated coefficient of the local Gini coefficient on meritocratic beliefs is positive and statistically significant for respondents with household incomes below £20,000, a group that comprise approximately 25% of the sample. The estimated coefficient of income inequality is not distinguishable from zero for those with household incomes between £20,000 and £90,000 but becomes negative and statistically significant among respondents with household incomes greater than £90,000, a group that comprises approximately 3.4% of the sample. This suggests that high levels of income inequality are associated with weaker meritocratic beliefs among very high‐income respondents.

**FIGURE 3:**
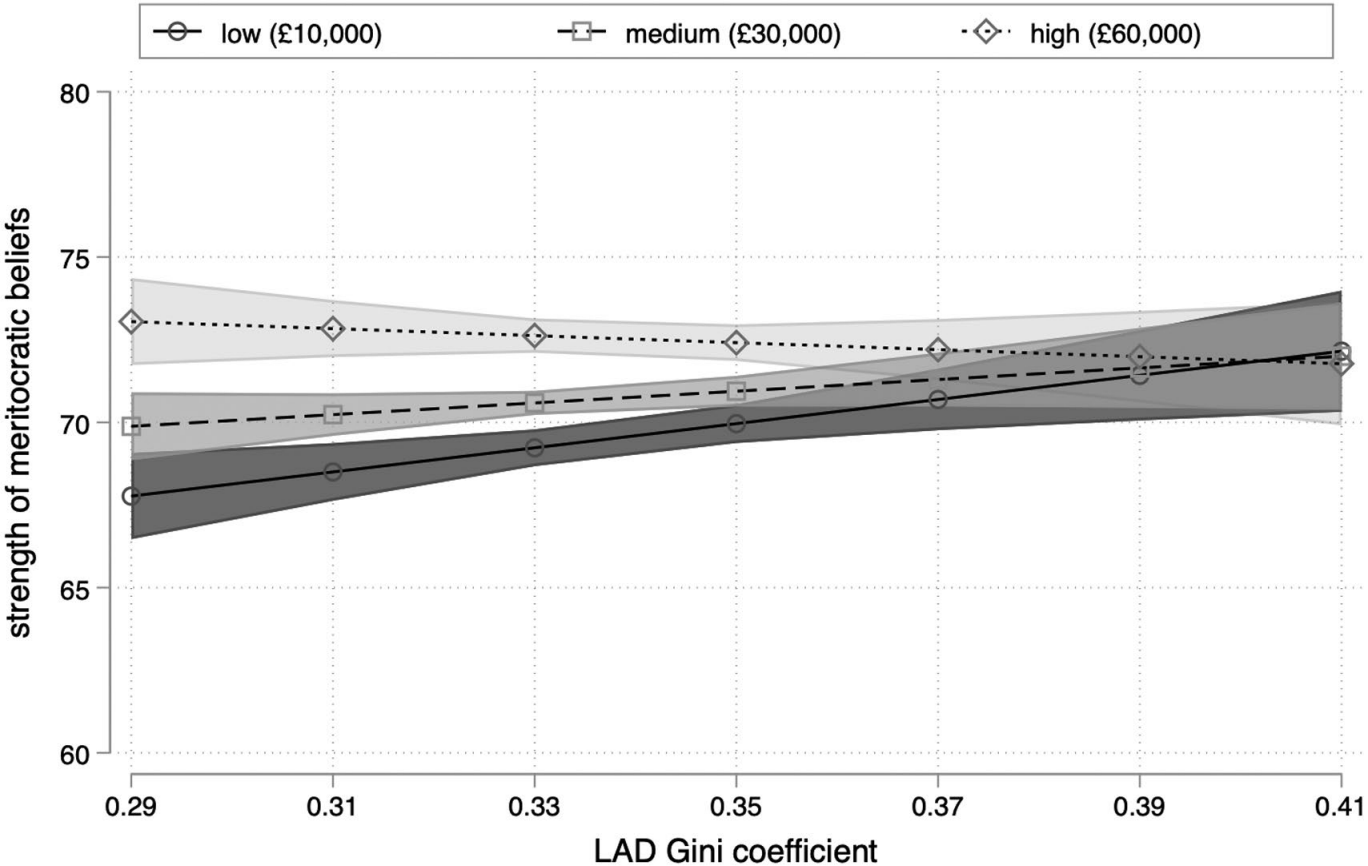
Predicted meritocratic beliefs by household income and level of inequality

This pattern can also be seen in Figure 3, which plots the predicted value of meritocratic beliefs among respondents with household incomes of £10,000, £30,000, and £60,000, respectively, values which approximate the 10th, 50th, and 90th percentile of the sample income distribution. Controlling for individual and contextual factors, respondents with household incomes of £10,000 report beliefs of 68 (±1) on the 100‐point scale in the most equal localities within England. This rises by 4 points to 72 (±2) in the most unequal localities, a difference that is greater than the average disparity between graduates and non‐graduates and those from white and ethnic minority backgrounds, and only slightly less than the difference between respondents who are unemployed and those who are not. Our findings thus support the analysis of Solt et al. (2016) rather than Newman et al. (2015): low‐income respondents are more likely to support (or less likely to reject) meritocracy if they live in more unequal localities.

### 4.2 Supplementary analysis: 80:20 ratio

**FIGURE 4:**
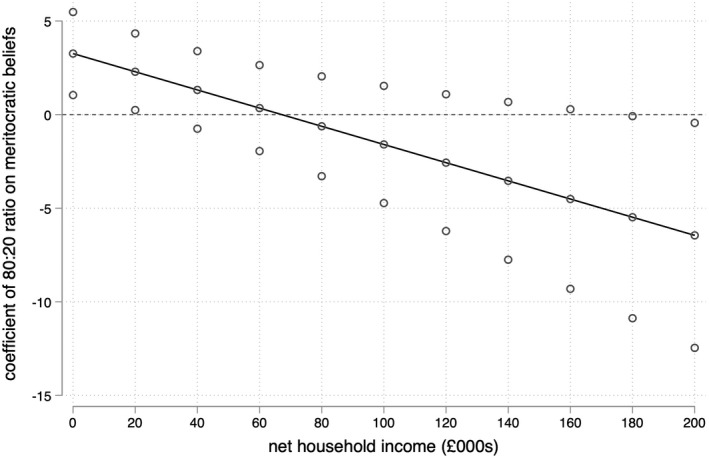
Coefficient of local income inequality on meritocratic beliefs by household income

**FIGURE 5:**
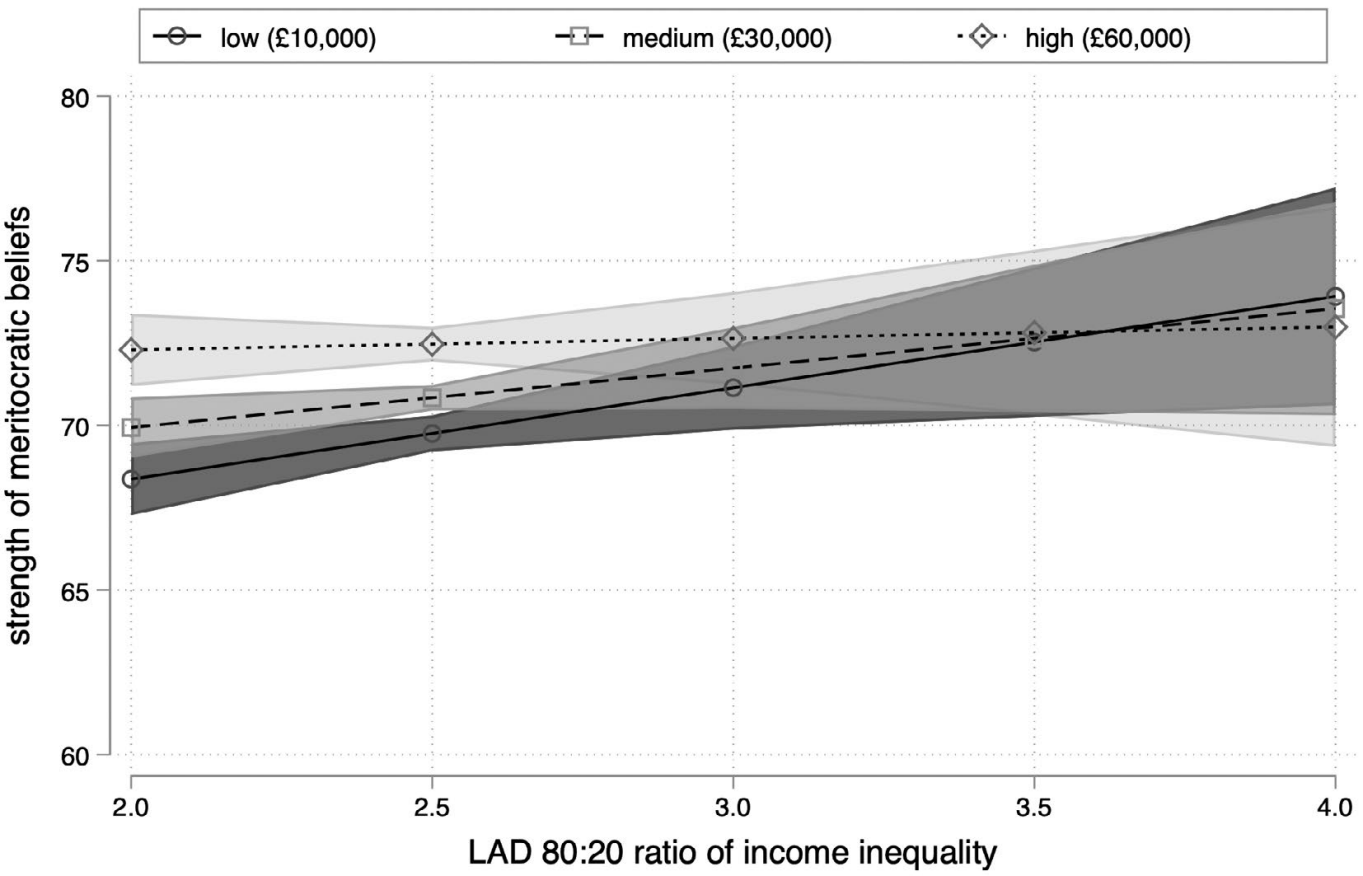
Predicted meritocratic beliefs by household income and level of inequality

Repeating Steps 1 and 2 with the 80:20 ratio of local income inequality rather than the Gini coefficient paints a similar but non‐identical picture. Against the expectations of Hypothesis 1, Model III in Table 2 shows that there is also no robust association between local income inequality as measured by the 80:20 ratio and meritocratic beliefs, all else being equal. The interaction term introduced in Model IV in Table 2 is again negative, yielding support for Hypothesis H2b and against Hypothesis H2a. This can be seen more clearly in Figures 4 and 5: that this alternative measure of local income inequality produces very similar predictions for low‐income (£10,000) respondents reinforces the idea that higher levels of inequality are associated with stronger meritocratic beliefs among those at the bottom of the income distribution.

Where findings using this alternative specification of income inequality differ is in relation to wealthy respondents. Figure 4 shows that the coefficient for the 80:20 ratio only obtains significance among respondents with household incomes greater than £180,000, a group that comprises just 0.5% of the sample. But while this implies that the strength (and robustness) of the apparent negative relationship between income inequality and meritocratic beliefs among the wealthiest individuals is sensitive to the definition of local income inequality, Figures 3 and 5 nonetheless display a common trend. Irrespective of how local income inequality is measured, the robust positive association between inequality and meritocratic beliefs among low‐income respondents means that an increase in local income inequality is associated with the convergence of meritocratic beliefs across income lines.

### 4.3 Sensitivity analysis

**FIGURE A3:**
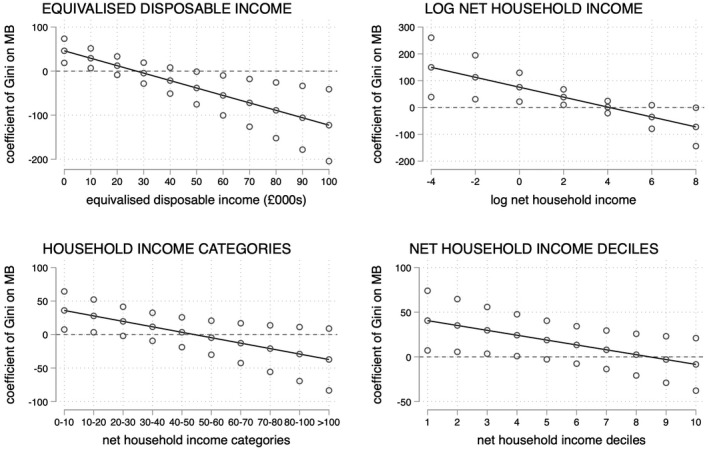
Coefficients of local income inequality on meritocratic beliefs by household income—alternative household income specifications

**FIGURE A4:**
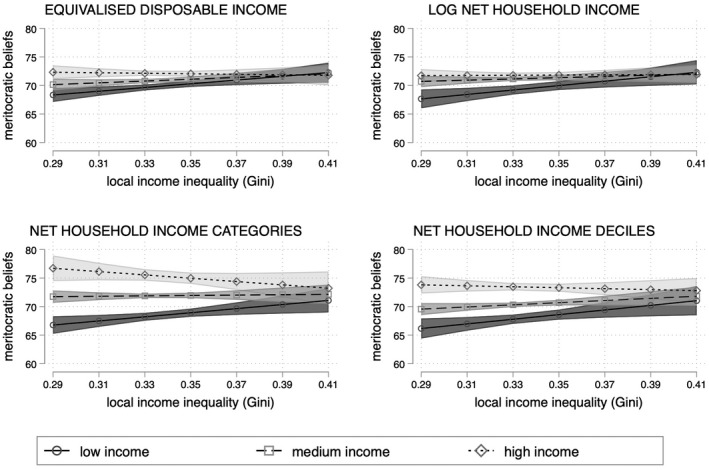
Predicted meritocratic beliefs by household income and level of income inequality—alternative household income specifications

**FIGURE A5:**
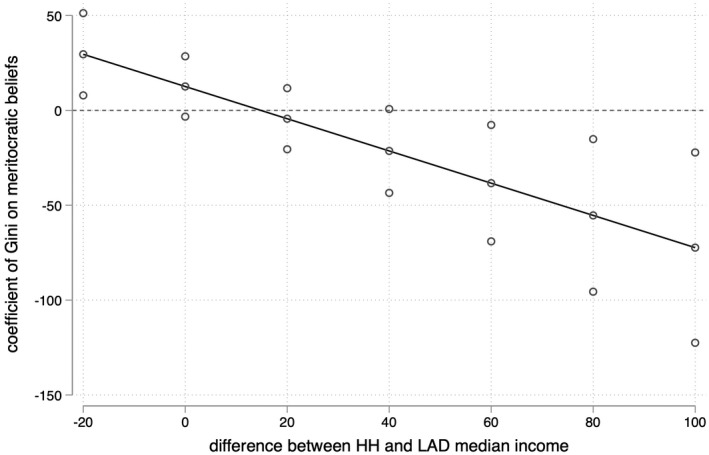
Coefficients of local income inequality on meritocratic beliefs by the gap between household and median LAD income

**FIGURE A6:**
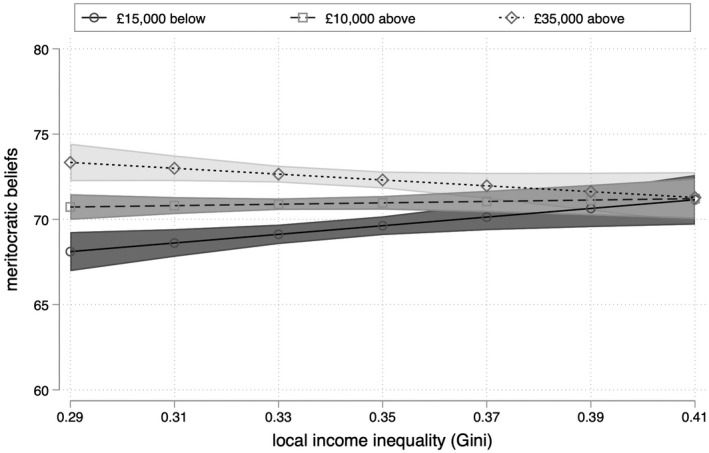
Predicted meritocratic beliefs by the gap between household and LAD median income and level of inequality

We conduct a number of additional analyses in order to probe the robustness of our findings, the results of which are displayed in the Appendix. Findings are robust to several alternative specifications of household income (Tables A3  and A4 and Figures A3 and A4) and are also unchanged when using a three‐level model to account for the household structure of the UKHLS survey (Table A5) and an alternative model which recognizes local variation in the value of absolute net household income (Table A6 and Figures A5 and A6). Findings are also robust to two alternative specifications of the duration of residence in the current Local Authority District^11^ that are designed to rule out the possibility that our results are driven by selective migration by people with optimistic tendencies (Table A7).

**FIGURE A7:**
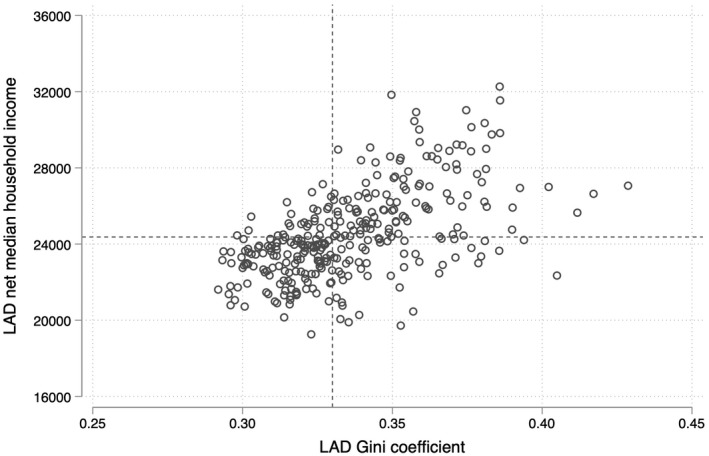
Relationship between LAD inequality and median household income

**FIGURE A8:**
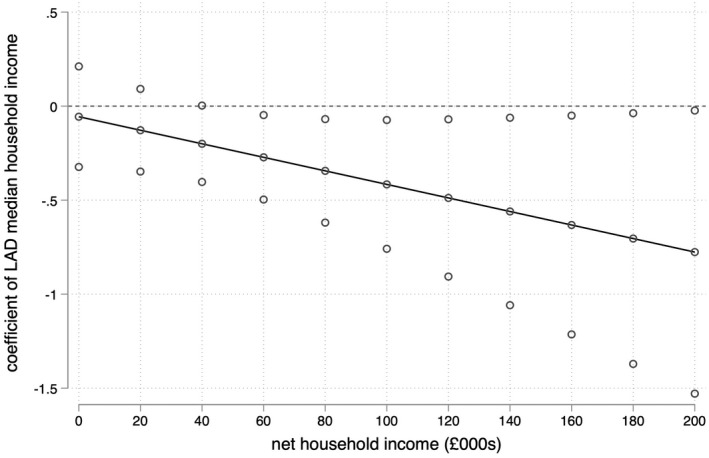
Coefficients of LAD median income on meritocratic beliefs by household income

Since more unequal localities tend to be more prosperous localities (Rae & Nyanzu, 2019a) and the Gini coefficient and median household income correlate at 0.56 at the LAD level, we also undertake additional analysis to try and ascertain whether it is exposure to inequality rather than exposure to affluence that really drives our results. We do this by interacting individual household income with LAD median household income rather than the LAD Gini coefficient: results (Table A6 and Figures A7 and A8 in the Appendix) show that there is no robust interaction between individual income and area wealth, controlling for the effect of the LAD Gini coefficient. We take this to mean that it is exposure to economic contrasts that play the more important role in producing the paradox of local inequality, though the correlation between area wealth and inequality is such that it is impossible to fully disentangle these effects.

Our conclusions therefore remain as before: local income inequality does not have a robust effect on the meritocratic beliefs of the sample as a whole, but there is a small but robust positive association between daily exposure to high levels of local income inequality and meritocratic beliefs among low‐income respondents.

## 5 DISCUSSION

Two sets of implications emerge from our analysis. First, the fact that local income inequality does not have a robust main effect on belief in meritocracy in England casts doubt on the social and spatial distancing explanation of the “paradox of (national) inequality” proposed by Mijs (2020). This explanation stresses the importance of interaction with and exposure to the economic other for illuminating the structural sources of inequality. Such interaction (be it in the form of direct contact or indirect exposure to symbols of poverty and affluence) is likely to be more frequent in unequal localities, which suggests that there should be an inverse relationship between local income inequality and the strength of individual meritocratic beliefs. That we cannot identify such a relationship in England—at least at the scale of Local Authority Districts—indicates that the net effect of contact and interaction across economic fault lines is not the illusion‐shattering one Mijs (2020) proposes.

This finding also means we should only cautiously use explanations that see belief in meritocracy as a conscious and explicit type of knowledge. Attitudes toward social status, social value, and individual merit are not purely cognitive and conscious phenomenon. These attitudes are not merely the result of the diffusion of information and the knowledge about theories of inequality. They probably also involve a sociopsychological comparison and justification dynamic. Principles of equality and fairness are likely to be embedded in how people perceive the social structure, how they situate themselves within social structures, and what prospect of future improvement they perceive to be realistic. More generally, this finding raises questions for studies comparing belief in meritocracy over time and between countries. If these meritocratic beliefs cannot be explained by patterns of spatial and social distancing, then we need to develop convincing alternative explanations.

Second, the fact that we largely replicate Solt et al.'s (2016) findings provides further indication that the theory of activated class conflict proposed by Newman et al. (2015) is flawed. In common with Solt et al. (2016), we find a small but robust positive association between daily exposure to higher levels of local income inequality and meritocratic beliefs among low‐income respondents in England. This association holds irrespective of whether local income inequality is measured via the standard Gini coefficient or our alternative 80:20 measure. Findings for wealthier individuals are more mixed, but the upshot of the positive effect of income inequality among low‐income respondents and the negative or noneffect among high‐income individuals is that the meritocratic beliefs tend to converge across income lines, as local‐income inequality rises.

Is the relative power theory explanation of this paradox of local inequality adequate? Since the lead author (Solt, 2014; Solt et al., 2011) has previously argued in cross‐national research that *greater inequality provides richer individuals with the motive and the means to disseminate religion more widely throughout their societies* (2011, pp. 447–448), the application of relative power theory to meritocratic beliefs can be seen as a straightforward substitution in two respects. First, meritocracy for religion, which is also held to be a system justifying tool of social control for the wealthy and a source of comfort for the poor. Second, of the local for the national, with the wealthy having more power to spread beneficial ideologies in highly unequal localities.

While there are some interesting parallels between meritocratic and religious belief systems in terms of the promise of future reward, the substitution of the local for the nation is more problematic. At the national level, it is at least plausible that elite capture of media and political channels facilitates the promulgation of meritocratic ideology. However, the channels of control and mechanisms of diffusion at the local level are rather less obvious and are left entirely unspecified by Solt et al. (2016). This raises the question of whether high levels of local income inequality really *provides higher‐income people with more resources to spread their views in the public sphere while depriving poorer people to a greater degree of the resources needed to resist these efforts* (Solt et al., 2016, p. 2) or whether the contact and interaction‐based mechanism proposed by Mijs (2020) and Newman et al. (2015) are rather more plausible.

Leaning strongly toward the interaction and exposure mechanism, we propose a system justification theory‐based explanation of the paradox of local inequality (Jost, 2020; Jost et al., 2004; Jost & Banaji, 1994; Jost & Hunyady, 2003). System justification theory maintains that people have an inherent subconscious need *to imbue the status quo with legitimacy and to see it as good, fair, natural, desirable* (Jost et al., 2004, p. 887), irrespective of whether the status quo is personally advantageous or disadvantageous. For those at the bottom of the status hierarchy, this theory is usually applied in a defensive sense, with accounts emphasizing the palliative function of the system justifying ideologies such as meritocracy. However, we are inclined toward the more positive slant offered by McCoy et al. (2013, p. 308), who observe that *belief in meritocracy may pose a benefit to the self‐esteem of members of low status groups because it is consistent with the perception that advancement is possible*. For low‐income respondents, who regularly see and/or interact with the economic other, we maintain that meritocratic ideology serves a dual function. Though it legitimates their current position at the bottom of the status hierarchy, it also offers the promise of future advancement, thereby transforming “have nots” into “‘soon to haves” (McCoy et al., 2013).

We attempt to substantiate this claim by undertaking an additional analysis of the relationship between local income inequality (measured as the Gini coefficient) and a concept that can offer insight into the validity of the future advancement hypothesis: income satisfaction. Intuitively, one would expect income satisfaction among low‐income respondents to decline with rising income inequality and exposure to the more affluent; if it does not, then we take this as evidence for the future advancement hypothesis. To enhance comparability with our measure of meritocratic beliefs, we first rescale the original 7‐point Likert response scale to a 0–100 score, where 0 denotes completely dissatisfied and 100 completely satisfied. We then run additional linear multilevel models of income satisfaction, using exactly the same set of covariates employed in our analysis of meritocratic beliefs.

**FIGURE 6:**
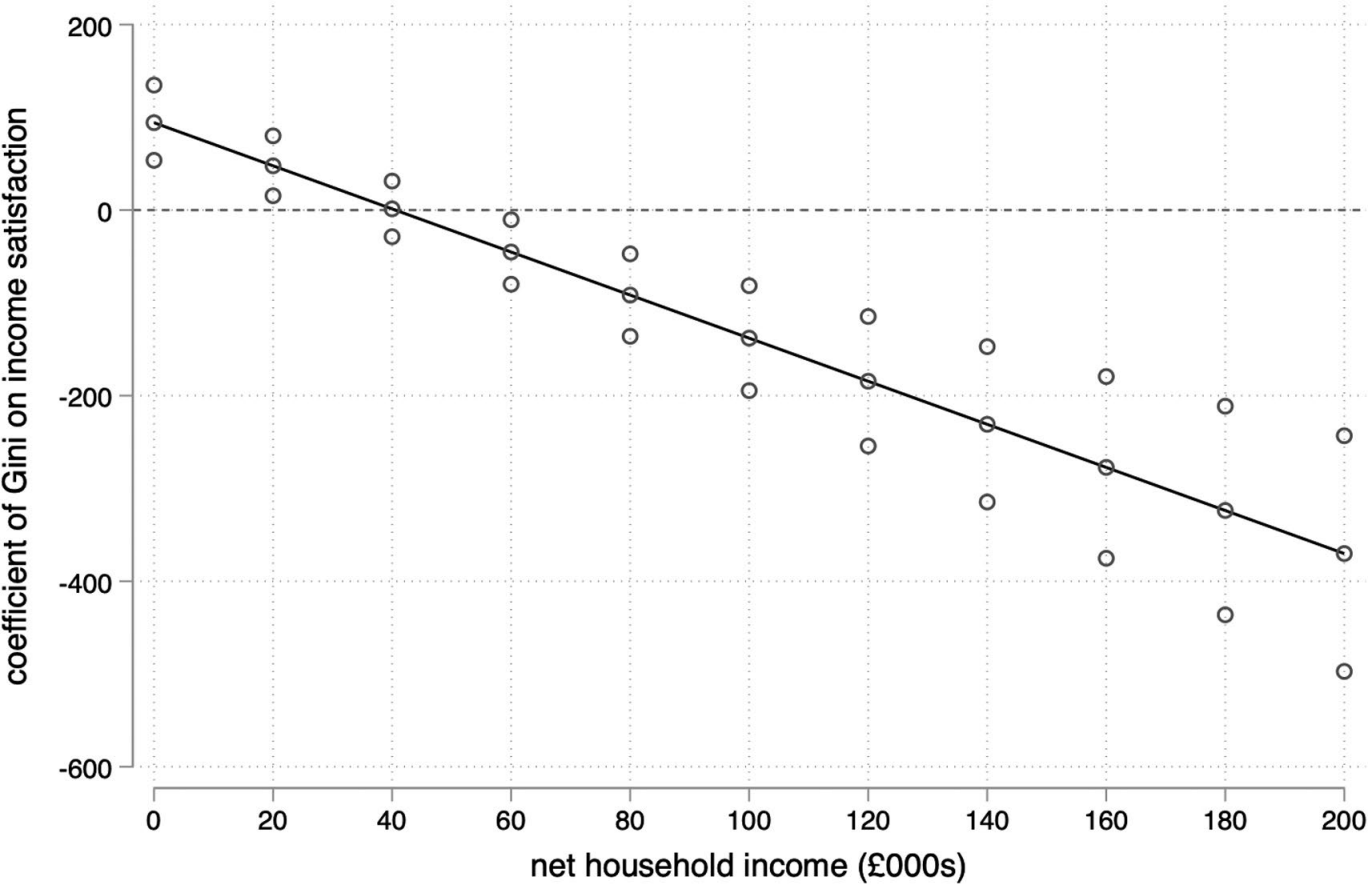
Coefficient of local income inequality on income satisfaction by household income

**FIGURE 7:**
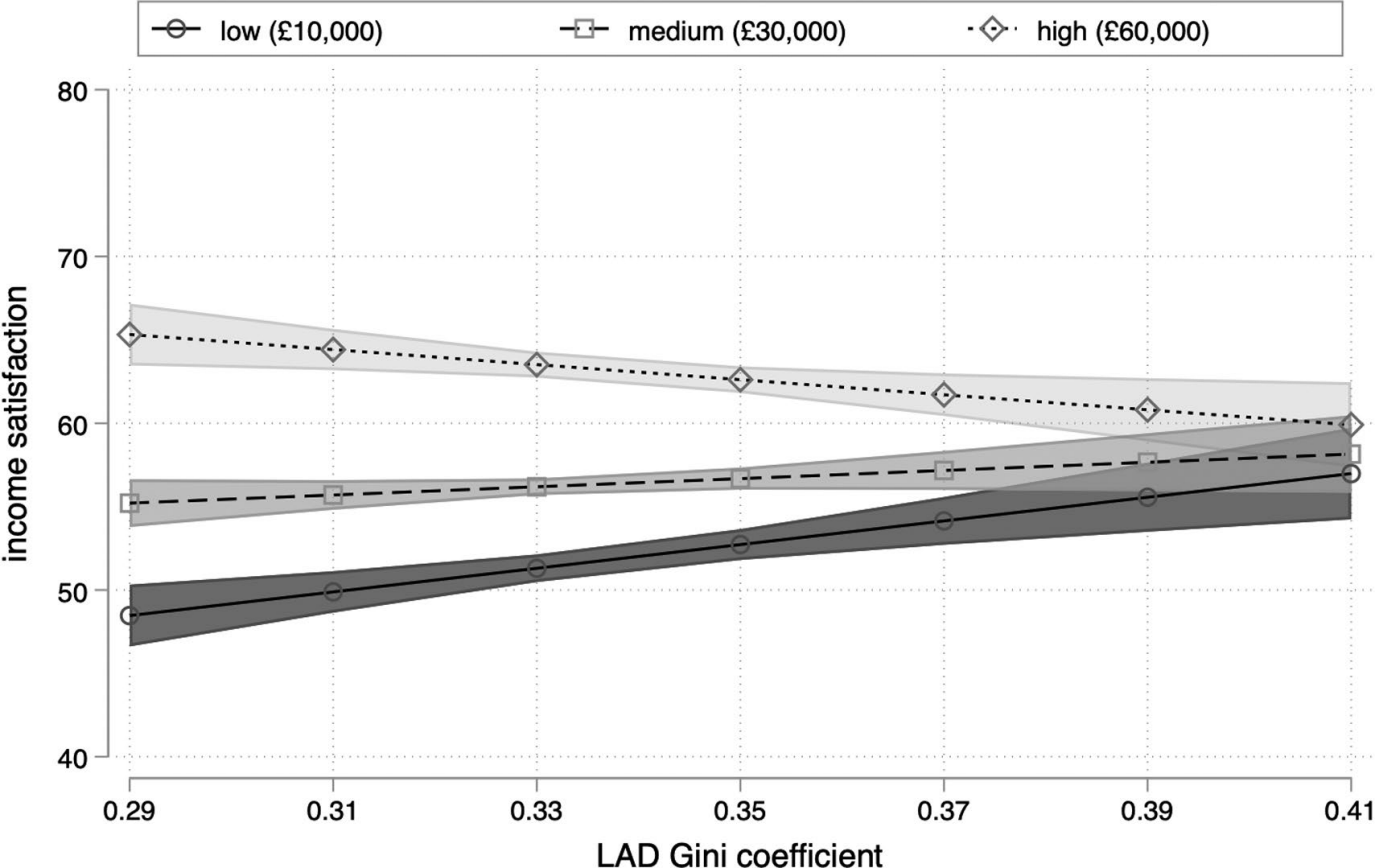
Predicted income satisfaction by household income and level of inequality

In line with the future advancement hypothesis, findings (displayed in Table 3 and Figures 6 and 7) are remarkably similar to those we obtain for meritocratic beliefs. Local income inequality has no main effect on income satisfaction, but Model VI shows that there is a robust positive association between local income inequality and the income satisfaction of respondents at the bottom of the income distribution. All else being equal, respondents with household incomes of £10,000 report income satisfaction of 48 (±2) on a 100‐point scale in the most equal localities within England, rising to 57 (±3), respectively, in the most unequal localities.

**TABLE 3:** Linear mixed models of income satisfaction among UKHLS respondents

	(V)	(VI)
Fixed effects		
LAD level		
LAD population (000s)	0.001	0.001
	(−0.003 – 0.004)	(−0.003 – 0.004)
LAD % BAME	−0.026	−0.017
	(−0.077 – 0.026)	(−0.075 – 0.040)
LAD median income (£000s)	0.069	0.043
	(−0.240 – 0.379)	(−0.292 – 0.377)
LAD inequality (Gini)	17.807	94.176***
	(−10.955 – 46.568)	(53.653 – 134.698)
Cross‐level interaction		
LAD Gini * income		−2.322***
		(−3.076 – −1.568)
Individual level		
Net HH income (£000s)	0.093***	1.010***
	(0.037 − 0.148)	(0.749 − 1.272)
Male	0.517	0.257
	(−0.252 – 1.286)	(−0.501 – 1.016)
Age	−1.100***	−1.133***
	(−1.219 – −0.980)	(−1.251 – −1.014)
Age^2^	0.012***	0.013***
	(0.011 − 0.013)	(0.012 − 0.014)
BAME	−4.843***	−4.616***
	(−6.369 – −3.317)	(−6.118 – −3.114)
Graduate	5.590***	4.352***
	(4.590 − 6.589)	(3.478 − 5.225)
Unemployed	−13.847***	−12.165***
	(−16.176 – −11.517)	(−14.313 – −10.017)
Belongs to a religion	0.420	0.237
	(−0.480 – 1.320)	(−0.647 – 1.121)
Political leanings	2.755***	2.252***
	(1.755 − 3.756)	(1.293 − 3.211)
	−2.330***	−2.190***
	(−3.404 – −1.256)	(−3.249 – −1.131)
Lives in the same LAD	0.045	−0.444
	(−1.561 – 1.652)	(−2.057 – 1.168)
Constant	65.603***	36.372***
Random effects		
Income		0.004
LAD (constant)	5.245	4.111
Residual	747.563	729.406
Number of respondents	**24,936**	**24,936**
Number of LADs	**315**	**315**

*Source*: UKHLS Wave 5. Confidence intervals in parenthesis.

*** *p* < .001

** *p* < .01

* *p* < .05.

Bold value indicates sample N.

Although a full test of the paradox of local inequality would require longitudinal data, the fact that low‐income respondents in highly unequal localities tend to express notably higher levels of satisfaction with their own (low) income, as well as stronger meritocratic beliefs suggests that these beliefs may well be turned toward the future. For the most economically vulnerable members of society, it is at least plausible that regular direct and indirect contact with others “who have made it,” coupled with the higher levels of economic dynamism in more unequal localities (Lee et al., 2016), strengthens belief in the possibility of overcoming the present economic situation with hard work. We hope that it be will possible to test this hypothesis more fully in the future.

### 5.1 Gaps and remaining questions

Though our findings closely align with those of Solt et al. (2016) in the United States, there remain a number of unanswered questions. First, it is not clear whether the paradox of local inequality is widespread or specific to these two countries or the Anglosphere more generally. The United Kingdom is relatively similar to the United States in terms of its Anglo‐Saxon capitalist model, liberal welfare state regime, and more individualistic cultural scripts (Andersen et al., 2021), traits also shared by Australia, Canada, Ireland, and New Zealand. Future research could usefully investigate whether the same seemingly paradoxical pattern emerges in more and less similar settings. This would help shed light on the generalisability of our findings and the mechanisms underlying the trends identified in the US and the UK.

Second, there is the question of the precise scale at which the effects of local income inequality play out and which dimension or dimensions of local inequality are most formative. We follow Newman et al. (2015) and Solt et al. (2016) in selecting local government districts as our analytical unit and the Gini coefficient as our main measure of income inequality in order to increase the comparability of our findings, but both decisions have shortcomings. The use of administrative units that do not reflect real social or economic geography is likely to be a particularly problematic in the case of people who live on or close to a unit border and those who commute long distances: these individuals may well be influenced by neighboring areas in a way our modeling strategy (and that of previous authors) does not account for. Furthermore, similar local Gini coefficients (or 80:20 ratios) can reflect different overall income distributions and spatial concentrations of poverty and affluence. It may be that living in a highly unequal locality in which pockets of advantage and disadvantage are highly spatially concentrated makes inequality more salient than living in one in which rich and poor live side‐by‐side, with important consequences for how individuals construct their understanding of inequality and their own position in society (Bottero, 2019). Further research is needed to understand both spheres of inequality influence and the role of different spatial segregation dynamics within these spheres of influence.

Third, it is not clear how the local environment interacts with other sources of information about inequality and opportunity. People acquire (often contradictory) information from multiple sources and life domains, including friends and family, news media, and even public entertainment (Kim, 2021). Our understanding of the relative role of the local environment vis‐à‐vis these other sources of information is currently very limited.

## 6 CONCLUSIONS

In this article, we contribute to a recent debate by undertaking the first assessment of the relationship between local income inequality and meritocratic beliefs outside the United States, using data from the UK Household Longitudinal Study. We find that the positive relationship between country‐level income inequality and meritocratic beliefs identified by Mijs (2020) does not translate straightforwardly below country level: there is no meaningful association between LAD‐level income inequality and meritocratic beliefs in England across the sample as a whole. But there is a robust—and somewhat paradoxical—positive relationship between high local income inequality and meritocratic beliefs among a specific subset of the population: those with the lowest incomes. We interpret this curious finding as evidence that belief in meritocracy can serve as an important tool of psychological resilience for low‐income individuals who regularly come into contact—directly or indirectly—with others who are better off than they are. Though such contact may highlight an individual's current lowly position in the income distribution, it can also make escape via upward income mobility more conceivable and plausible.

In combination, our results have far‐reaching implications for a collective understanding of how income inequality affects individual well‐being and whether such inequality is self‐correcting. If higher levels of local income inequality did depress belief in the core premise of the western democratic system—either among the population as a whole or among the most economically vulnerable—then two things would follow. While there would be reasons to worry about the psychological effects of high levels of local income inequality, we could also expect greater bottom‐up demand for redistribution to emerge from unequal localities and self‐correction processes to kick in. But since we find no general association, and since living in places of higher income inequality is actually associated with higher levels of meritocratic belief (and income satisfaction) among those who are most economically vulnerable within England, the converse holds. There is less reason to worry about the unequal psychological effects of income inequality but there is also little prospect of system correction. Low‐income respondents are unlikely to embark on political action intended to overthrow the unequal status quo if they endorse meritocratic ideology with the same fervor as their much wealthier counterparts.

In order to corroborate—or refute—this interpretation of the spatial and social distribution of belief in meritocracy, future research should dig deeper into how belief in meritocracy is linked to other attitudes, dispositions, and values. For instance, do low‐income individuals and families in highly unequal areas have a distinctive orientation toward the future? Is their belief integrated into a typical configuration of dispositions or attitudes? But as highlighted above, researchers must also dig deeper into the meaning of living in an unequal context: what does it mean to individuals, and what are the features of these unequal contexts in terms of neighborhood networks, local public spheres, and social interaction more generally?

### ACKNOWLEDGMENTS

We thank the UK Data Service for their assistance in obtaining the special licence required to access restricted versions of Understanding Society used in this paper, and participants of the UNIL Parcours de vie et inégalités sociales seminar and the ECSR Annual Conference 2021 for their helpful comments and ideas. We would also like to express our thanks to the BJS editorial team and two anonymous reviewers for the pertinent and helpful feedback and the constructive spirit of challenge and engagement, which were both greatly appreciated. Open access funding provided by Universite de Lausanne.

### REPLICATION STATEMENT

Replication Stata syntax, which includes links to the individual and LAD‐level data employed in the analysis have been archived at: https://osf.io/svwn2/.

## Figures and Tables

**FIGURE A9:**
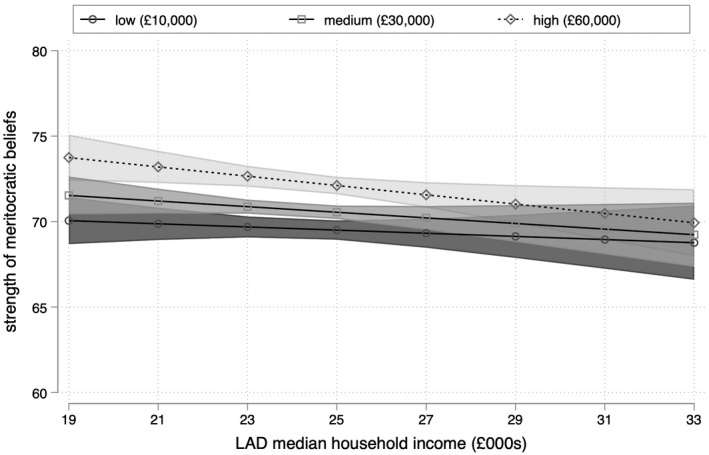
Predicted meritocratic beliefs by household income and LAD median income

## Data Availability

Data subject to third‐party restrictions. The individual‐level UKHS data that support the findings of this study are available from the UK Data Archive (SN 6614; SN 6666, SN 6931). Restrictions apply to the availability of these data, which were used under license for this study. LADAUTHOR: As per journal style, please provide missing Acknowledgements section.‐level data sources are documented and linked to in the article text as well as in the replication syntax described below.

## Endnotes

^1^
The errors documented by Newman et al. relate to the analysis of the nonwhite sample (Newman et al., 2016a) and the use of an R package that is no longer supported (Newman et al., 2016b), but they claim that their results remain unchanged once these errors have been corrected. Solt et al. (2016) state in a footnote, “Newman et al. were offered the opportunity to respond to this article in print … but declined to do so.”

^2^
The meaning of inequalities and the meaning of an individual's social position is always constructed relative to others (Bottero, 2019). Therefore, what matters for individual attitudes to meritocracy might be not only the very local context but also the larger regional context in which this local context is embedded.

^3^
According to Newman et al. (2015), African Americans are less sanguine about the existence of economic opportunities, have different sensitivities concerning their personal economic situation and local economic conditions.

^4^
For confidentiality reasons, locality data are not made publicly available but are accessible via a UK Data Archive Special Licence.

^5^
The 6791 English Middle‐layer Super Output Areas (MSOAs) are small statistical units notionally defined as areas with populations of 4000 households and 7500 residents.

^7^
The item we use as our dependent variable (originally named *delay10*) is asked at the end of a battery of survey items about delayed gratification in Wave 5 of UKHLS. To ensure that responses are not affected by anchoring bias, we cross‐check the correlations between our dependent variable and the other nine delayed gratification items. Results, displayed in Tables A1 and A2 in the Appendix, show that none of the other items correlate strongly with the item we use as our dependent variable (or with each other), which reduces concerns about the influence of the preceding delayed gratification items.

^8^
Atlas of Inequality (Rae & Nyanzu, 2019a, 2019b) data are available from https://github.com/alasdairrae/alasdairrae.github.io/tree/master/atlasofinequality/data.

^9^
The Gini coefficient is usually constructed based on individual rather than household income. We use household‐based Gini estimates based on availability and our use of household income data.

^10^
ONS Admin‐based income statistics, England and Wales: tax year ending 2016; data are available from https://bit.ly/3rCqF7v.

^11^
These are variously (a) a binary variable, which takes value 1 if respondents are deemed to be living in the same place they were born in if they live in a Local Authority District that corresponds to their given place of birth, and 0 otherwise (see Lee et al., 2018 for more details on variable construction) and (b) a continuous indicator of the number of years respondents have been living at their current address.

## APPENDIX A

1) LAD Units and Metrics

2) Dependent Variable

Our dependent variable (*delay10*, “I have always felt like my hard work would pay off in the end”) is part of a one‐off module about delayed gratification in Wave 5 of Understanding Society, which also solicits responses to items such as “I would have a hard time sticking with a special, healthy diet” and “I cannot motivate myself to accomplish long‐term goals.”

Since *delay10* is the last of the 10 survey items posed to respondents, we check the correlations between our dependent variable and the other delayed gratification survey items to ensure that responses to our dependent variable do not suffer from substantial anchoring bias. The correlations displayed in Tables A1 and A2 show that the other items do not correlate strongly with *delay10* responses or with each other. That only one of the items (*delay8*, “I have always tried to eat healthy because it pays off in the long run”) correlates with our dependent variable at above 0.3 reduces concerns about conceptual overlap and construct validity.

**TABLE A1:** Correlations of UKHLS Wave 5 delayed gratification items with our dependent variable

Delay10	I have always believed my hard work will pay off in the end	1.000
**Delay1**	I would have a hard time sticking with a special, healthy diet	−0.054
**Delay2**	I try to spend money wisely	0.230
**Delay3**	I have given up physical pleasure or comfort to reach my goals	0.060
**Delay4**	I try to consider how my actions will affect other people in the long term	0.251
**Delay5**	I cannot be trusted with money	−0.099
**Delay6**	I do not consider how behavior affects other people	−0.058
**Delay7**	I cannot motivate myself to accomplish long‐term goals	−0.195
**Delay8**	I have always tried to eat healthy because it pays off in the long run.	0.316
**Delay9**	When faced with a physically demanding chore, I always tried to put off doing it	−0.090

Bold value indicates sample N

**TABLE A2:** UKHLS Wave 5 delayed gratification module—survey item correlations

	Delay10	Delay1	Delay2	Delay3	Delay4	Delay5	Delay6	Delay7	Delay8	Delay9
**Delay10**	1.000									
**Delay1**	−0.054	1.000								
**Delay2**	0.230	−0.002	1.000							
**Delay3**	0.060	0.103	0.093	1.000						
**Delay4**	0.251	0.013	0.314	0.092	1.000					
**Delay5**	−0.099	0.119	−0.257	0.059	−0.152	1.000				
**Delay6**	−0.058	0.095	−0.098	0.067	−0.236	0.268	1.000			
**Delay7**	−0.195	0.203	−0.101	0.043	−0.105	0.260	0.276	1.000		
**Delay8**	0.316	−0.239	0.262	0.025	0.205	−0.094	−0.041	−0.097	1.000	
**Delay9**	−0.090	0.183	−0.076	0.075	−0.058	0.186	0.160	0.307	−0.069	1.000

Bold value indicates sample N

3) Sensitivity Analysis

We conduct a number of additional analyses in order to probe the robustness of our findings. First, to ensure that results are not sensitive to the specification of household income, we test a number of alternative income measures including equivalized disposable income (using OECD equivalization weights), logged net household income, net household income categories, and net household income deciles. For all four additional specifications of household income, results (reported in Tables A3 and A4) are substantively very similar to our chosen measure, that of raw net annual household income.

**TABLE A3:** Testing alternative income specifications: main effect of income inequality

	(1) Equivalized disposable income (£000)	(2) Log net household income	(3) Household income categories	(4) Household income deciles (national)
Fixed effects				
LAD level				
LAD population (000s)	0.001	0.001	0.001	0.001
	(−0.001 – 0.003)	(−0.001 – 0.003)	(−0.001 – 0.003)	(−0.001 – 0.003)
LAD % BAME	−0.030	−0.031	−0.035*	−0.033
	(−0.070 – 0.009)	(−0.071 – 0.009)	(−0.075 – 0.004)	(−0.073 – 0.007)
LAD median Income (£000s)	−0.139	−0.154	−0.203**	−0.198**
	(−0.334 − 0.056)	(−0.350 − 0.042)	(−0.400 − −0.006)	(−0.393 − −0.003)
LAD Gini	11.534	13.179	10.123	12.115
	(−9.144 − 32.212)	(−7.673 − 34.032)	(−10.890 − 31.137)	(−8.741 − 32.970)
Cross‐level interaction				
LAD inequality * income				
Individual level				
Income measure	0.031*	1.077***	0.826***	0.599***
	(−0.001 − 0.063)	(0.611 − 1.543)	(0.649 − 1.002)	(0.464 − 0.735)
Male	−0.409	−0.443	−0.541	−0.553
	(−1.112 − 0.294)	(−1.147 − 0.260)	(−1.247 − 0.165)	(−1.259 − 0.153)
Age	−0.200***	−0.201***	−0.210***	−0.220***
	(−0.308 − −0.092)	(−0.310 − −0.093)	(−0.318 − −0.102)	(−0.329 − −0.112)
Age^2^	0.002***	0.002***	0.003***	0.003***
	(0.001 − 0.003)	(0.001 − 0.004)	(0.002 − 0.004)	(0.002 − 0.004)
BAME	3.002***	3.015***	3.160***	3.100***
	(1.938 − 4.065)	(1.937 − 4.093)	(2.076 − 4.244)	(2.019 − 4.182)
Graduate	2.719***	2.622***	2.210***	2.235***
	(1.985 − 3.453)	(1.902 − 3.342)	(1.498 − 2.923)	(1.528 − 2.941)
Unemployed	−4.899***	−4.425***	−4.086***	−3.904***
	(−6.765 − −3.033)	(−6.303 − −2.548)	(−5.957 − −2.215)	(−5.788 − −2.020)
Belongs to a religion	1.943***	1.868***	1.813***	1.798***
	(1.185 − 2.701)	(1.105 − 2.632)	(1.053 − 2.574)	(1.035 − 2.561)
Political leanings	2.567***	2.536***	2.332***	2.365***
	(1.745 − 3.388)	(1.720 − 3.352)	(1.515 − 3.148)	(1.546 − 3.184)
	−0.588	−0.569	−0.496	−0.493
	(−1.502 − 0.326)	(−1.485 − 0.347)	(−1.420 − 0.428)	(−1.415 − 0.429)
Lives in the same LAD	−1.450***	−1.684***	−1.784***	−1.831***
	(−2.516 − −0.385)	(−2.751 − −0.617)	(−2.853 − −0.716)	(−2.901 − −0.761)
			
Constant	72.024***	68.878***	71.535***	70.577***
Random effects				
Income				
LAD (constant)	1.380	1.369	1.325	1.347
Residual	555.245	554.948	553.206	553.401
Number of respondents	**24,943**	**24,943**	**24,943**	**24,943**
Number of LADs	**315**	**315**	**315**	**315**

*Source*: UKHLS Wave 5. Confidence intervals in parenthesis.

*** *p* < .001

** *p* < .01

* *p* < .05.

Bold value indicates sample N

**TABLE A4:** Testing alternative income specifications: conditional effect of income inequality

	(1) Equivalized disposable income (£000s)	(2) Log net household income	(3) Household income categories	(4) Household income deciles (national)
Fixed effects				
LAD level				
LAD population (000s)	0.001	0.001	0.001	0.001
	(−0.001 − 0.004)	(−0.001 − 0.003)	(−0.001 − 0.004)	(−0.001 − 0.004)
LAD % BAME	−0.031	−0.030	−0.034	−0.032
	(−0.071 − 0.010)	(−0.070 − 0.010)	(−0.074 − 0.007)	(−0.072 − 0.009)
LAD median Income (£000s)	−0.169*	−0.154	−0.199**	−0.201**
	(−0.369 − 0.031)	(−0.349 − 0.041)	(−0.396 − −0.002)	(−0.398 − −0.004)
LAD Gini	46.136***	75.801***	44.071***	46.050**
	(18.687 − 73.584)	(21.956 − 129.645)	(10.770 − 77.371)	(8.496 − 83.604)
Cross‐level interaction				
LAD Gini * income	−1.688***	−18.517**	−8.129**	−5.444**
	(−2.655 − −0.721)	(−33.290 − −3.744)	(−14.834 − −1.424)	(−10.623 − −0.266)
Individual level				
Income measure	0.670***	7.406***	3.601***	2.429***
	(0.332 − 1.009)	(2.269 − 12.544)	(1.295 − 5.907)	(0.668 − 4.190)
Male	−0.461	−0.459	−0.550	−0.556
	(−1.162 − 0.241)	(−1.161 − 0.244)	(−1.255 − 0.155)	(−1.260 − 0.149)
Age	−0.216***	−0.202***	−0.210***	−0.220***
	(−0.324 − −0.108)	(−0.310 − −0.093)	(−0.318 − −0.101)	(−0.329 − −0.111)
Age^2^	0.003***	0.002***	0.003***	0.003***
	(0.001 − 0.004)	(0.001 − 0.004)	(0.002 − 0.004)	(0.002 − 0.004)
BAME	3.121***	2.992***	3.136***	3.084***
	(2.042 − 4.200)	(1.914 − 4.070)	(2.053 − 4.219)	(2.004 − 4.164)
Graduate	2.328***	2.606***	2.198***	2.242***
	(1.613 − 3.042)	(1.888 − 3.325)	(1.487 − 2.909)	(1.538 − 2.947)
Unemployed	−4.412***	−4.383***	−4.047***	−3.909***
	(−6.257 − −2.567)	(−6.265 − −2.502)	(−5.916 − −2.177)	(−5.795 − −2.023)
Belongs to a religion	1.919***	1.866***	1.808***	1.791***
	(1.165 − 2.673)	(1.103 − 2.630)	(1.049 − 2.568)	(1.031 − 2.552)
Political leanings	2.425***	2.542***	2.332***	2.360***
	(1.613 − 3.237)	(1.728 − 3.357)	(1.515 − 3.148)	(1.545 − 3.174)
	−0.534	−0.549	−0.484	−0.490
	(−1.449 − 0.382)	(−1.466 − 0.367)	(−1.407 − 0.439)	(−1.408 − 0.427)
Lives in the same LAD	−1.412**	−1.699***	−1.816***	−1.854***
	(−2.486 − −0.337)	(−2.767 − −0.630)	(−2.884 − −0.749)	(−2.924 − −0.785)
Constant	60.152***	47.500***	59.877***	59.290***
Random effects				
Income	0.002	0.001	0.020	0.001
LAD (constant)	0.680	1.355	1.051	0.914
Residual	553.153	554.766	552.879	552.691
Number of respondents	**24,943**	**24,943**	**24,943**	**24,943**
Number of LADs	**315**	**315**	**315**	**315**

*Source*: UKHLS Wave 5. Confidence intervals in parenthesis.

*** *p* < .001

** *p* < .01

* *p* < .05.

Bold value indicates sample N

In all cases, Table A3 shows that local income inequality (measured via the Gini coefficient) does not have a robust main effect on meritocratic beliefs: there is a small positive association but this is not significant at conventional statistical thresholds. However, the introduction of an interaction term between the local Gini coefficient and the four alternative measures of income in Table A4 again yields support for the conditional effects hypothesis. For all four additional specifications of household income, the interaction term between income and inequality is robust and negative and Figure A3 shows the coefficient of Gini on income is positive and robust among low‐income respondents across specifications, indicating that low‐income respondents are consistently more likely to hold stronger meritocratic beliefs if they live in more unequal areas. Findings among high‐income respondents are again somewhat varied, but Figure A4 shows that the trend of convergence across income groups with higher levels of inequality is consistent across the different specifications of household income.

Second, to account for the household structure of the UKHLS survey and the possible clustering of responses among individuals in the same households, we employ a three‐level model in which individuals are embedded first in households and then in Local Authority Districts. Since results (reported in Table A5) are substantively identical to those we obtain with a two‐level model, and since the mean number of observations per household is just 1.5, we select the two‐level model on the basis of parsimony.

Third, we specify an alternative model in which we explicitly recognise that identical household income does not necessarily mean identical relative status or affluence (Ogorzalek et al., 2020), owing to variation in the income distribution and costs of living across localities in England. By subtracting respondents' net household income from LAD median household income, dropping the LAD median income variable from our model and interacting local income inequality with the gap (positive or negative) between LAD median and net household income, we explore how local income inequality affects the meritocratic beliefs of respondents with incomes substantially below or above the local median. Findings, reported in Table A6, Figures A5 and A6 mirror our main results: respondents with household incomes substantially below the local median tend to profess stronger meritocratic beliefs if they live in more unequal areas.

**TABLE A5:** Three‐level linear mixed model of meritocratic beliefs

	LAD GINI	
	(1)	(2)
Fixed effects		
LAD level		
LAD population (000s)	0.001	0.001
	(−0.001 − 0.003)	(−0.001 − 0.003)
LAD % BAME	−0.028	−0.026
	(−0.066 − 0.009)	(−0.064 − 0.012)
LAD median Income (£000s)	−0.134	−0.142
	(−0.323 − 0.056)	(−0.331 − 0.048)
LAD inequality measure	7.843	43.156***
	(−12.599 − 28.284)	(16.751 − 69.560)
Cross‐level interaction		
LAD inequality * income		−1.024***
		(−1.505 − −0.542)
Individual level		
	0.027***	0.401***
	(0.007 − 0.047)	(0.232 − 0.571)
Male	−0.432	−0.474
	(−1.124 − 0.259)	(−1.162 − 0.213)
Age	−0.169***	−0.180***
	(−0.275 − −0.064)	(−0.285 − −0.074)
Age^2^	0.002***	0.002***
	(0.001 − 0.003)	(0.001 − 0.003)
BAME	3.139***	3.146***
	(2.078 − 4.200)	(2.083 − 4.210)
Graduate	2.493***	2.284***
	(1.758 − 3.229)	(1.567 − 3.002)
Unemployed	−4.351***	−4.061***
	(−6.218 − −2.485)	(−5.910 − −2.211)
Belongs to a religion	1.992***	1.950***
	(1.241 − 2.742)	(1.202 − 2.697)
Political leanings	2.486***	2.400***
	(1.694 − 3.277)	(1.614 − 3.187)
	−1.006**	−0.974**
	(−1.858 − −0.153)	(−1.829 − −0.118)
Lives in the same LAD	−1.306**	−1.390**
	(−2.359 − −0.253)	(−2.451 − −0.329)
Constant	71.908***	59.444***
Random effects		
Income		0.001
LAD	0.001	0.001
HH	152.732	152.815
Residual	400.615	400.844
Number of respondents	**24,943**	**24,943**
Number of households	**16,226**	**16,226**
Number of LADs	**315**	**315**

*Source*: UKHLS Wave 5. Confidence intervals in parenthesis.

*** *p* < .001

** *p* < .01

* *p* < .05.

Bold value indicates sample N

**TABLE A6:** Alternative model of meritocratic beliefs—cluster‐mean centered household income

	LAD GINI	
	(1)	(2)
Fixed effects		
LAD level		
LAD population (000s)	0.002	0.002
	(−0.001 − 0.004)	(−0.001 − 0.004)
LAD % BAME	−0.021	−0.020
	(−0.055 − 0.014)	(−0.055 − 0.015)
LAD median income (£000s)		
	
LAD inequality (Gini)	2.644	12.582
	(−12.591 − 17.878)	(−3.317 − 28.481)
Cross‐level interaction		
LAD Gini * income gap		−0.849***
		(−1.377 − −0.321)
Individual level		
Income gap, (vs. LAD median income)	0.021**	0.351***
	(0.001 − 0.042)	(0.167 − 0.535)
Male	−0.427	−0.513
	(−1.136 − 0.282)	(−1.215 − 0.189)
Age	−0.197***	−0.208***
	(−0.305 − −0.089)	(−0.316 − −0.100)
Age^2^	0.002***	0.003***
	(0.001 − 0.003)	(0.002 − 0.004)
BAME	2.998***	3.080***
	(1.933 − 4.064)	(2.006 − 4.154)
Graduate	2.705***	2.307***
	(1.971 − 3.439)	(1.597 − 3.017)
Unemployed	−4.832***	−4.304***
	(−6.710 − −2.954)	(−6.166 − −2.442)
Belongs to a religion	1.901***	1.832***
	(1.136 − 2.665)	(1.075 − 2.590)
Political leanings	2.533***	2.366***
	(1.708 − 3.358)	(1.551 − 3.181)
	−0.580	−0.536
	(−1.495 − 0.335)	(−1.454 − 0.382)
Lives in the same LAD	−1.533***	−1.698***
	(−2.600 − −0.467)	(−2.765 − −0.631)
Constant	71.692***	68.108***
Random effects		
Income gap		0.001
LAD (constant)	1.402	1.293
Residual	554.197	552.150
Number of respondents	**24,943**	**24,943**
Number of LADs	**315**	**315**

*Source*: UKHLS Wave 5. Confidence intervals in parenthesis.

*** *p* < .001

** *p* < .01

* *p* < .05.

Fourth, we run additional analysis to test whether our results are driven by selective migration to more unequal Local Authority Districts by highly motivated individuals with more optimistic or ambitious dispositions, a possibility that could render the association between local income inequality and meritocratic beliefs among low‐income respondents spurious. While our main analysis includes a control for whether or not respondents have lived in the same Local Authority Distinct since entering UKHLS, this variable only captures recent geographical mobility and is therefore of limited usefulness, especially for new UKHLS entrants.

We address this in two ways. In a first step, we use coding developed by Lee et al. (2018) to construct an alternative binary measure of lifetime geographical immobility, where 1 denotes that UKHLS respondents lives in the same place in which they were born at the time of the survey and 0 denotes that they have moved elsewhere at some point during the life course. This variable is also imperfect because the age at which respondents who moved away from their place of birth is unknown. We therefore also construct a second continuous variable using data from UKHLS (and its predecessor the British Household Panel Study) based on when respondents moved to their current address. This continuous indicator of the number of years respondents have been living at their current address (which ranges from 0 to a maximum of 84 years) is also imperfect, since the majority of residential moves within England are across a small geographical distance (Coulter & Ham, 2013; Coulter et al., 2016; Langella & Manning, 2019). However, the fact that results of analysis using these alternative specifications of immobility in Table A7 produces almost identical results reduces concerns about the effects of selective migration before respondents entered the UKHLS survey.

**TABLE A7:** Linear mixed model of meritocratic beliefs—alternative immobility indicators

	LIFETIME IMMOBILITY		YEARS AT CURRENT ADDRESS	
	(1)	(2)	(3)	(4)
Fixed effects				
LAD level				
LAD population (000s)	0.002*	0.002*	0.001	0.001
	(−0.000 − 0.004)	(−0.000 − 0.004)	(−0.001 − 0.003)	(−0.002 − 0.004)
LAD % BAME	−0.043*	−0.042*	−0.032	−0.028
	(−0.091 − 0.005)	(−0.091 − 0.007)	(−0.074 − 0.011)	(−0.072 − 0.016)
LAD median income	−0.099	−0.119	−0.115	−0.138
	(−0.320 − 0.121)	(−0.344 − 0.105)	(−0.334 − 0.104)	(−0.360 − 0.085)
LAD inequality (Gini)	11.153	39.013**	13.347	46.662***
	(−13.693 − 36.000)	(6.328 − 71.697)	(−11.711 − 38.404)	(16.712 − 76.612)
Cross‐Level Interaction				
LAD Gini * Income		−0.774***		−0.945***
		(−1.330 − −0.218)		(−1.482 − −0.407)
Individual level				
Net household income (£000s)	0.027**	0.326***	0.023**	0.382***
	(0.004 − 0.050)	(0.133 − 0.519)	(0.002 − 0.044)	(0.195 − 0.570)
Male	−0.378	−0.467	−0.284	−0.367
	(−1.108 − 0.351)	(−1.189 − 0.254)	(−1.046 − 0.478)	(−1.122 − 0.387)
Age	−0.185***	−0.195***	−0.206***	−0.222***
	(−0.301 − −0.069)	(−0.311 − −0.079)	(−0.324 − −0.087)	(−0.339 − −0.104)
Age^2^	0.002***	0.002***	0.002***	0.003***
	(0.001 − 0.003)	(0.001 − 0.004)	(0.001 − 0.004)	(0.002 − 0.004)
BAME	2.222***	2.208***	2.830***	2.883***
	(0.718 − 3.726)	(0.705 − 3.710)	(1.765 − 3.895)	(1.819 − 3.947)
Graduate	2.365***	2.028***	2.626***	2.272***
	(1.580 − 3.150)	(1.265 − 2.791)	(1.871 − 3.382)	(1.535 − 3.009)
Unemployed	−5.254***	−4.726***	−4.839***	−4.317***
	(−7.297 − −3.210)	(−6.742 − −2.711)	(−6.792 − −2.886)	(−6.252 − −2.381)
Belongs to a religion	2.131***	2.078***	2.032***	1.969***
	(1.354 − 2.909)	(1.306 − 2.850)	(1.274 − 2.791)	(1.217 − 2.720)
Political leanings	2.655***	2.532***	2.532***	2.385***
	(1.799 − 3.511)	(1.678 − 3.386)	(1.679 − 3.385)	(1.542 − 3.228)
	−0.564	−0.520	−0.792*	−0.735
	(−1.567 − 0.438)	(−1.524 − 0.485)	(−1.726 − 0.141)	(−1.671 − 0.201)
Alt immobility indicator	−0.071	−0.020	−0.007	−0.011
	(−0.909 − 0.767)	(−0.857 − 0.817)	(−0.041 − 0.028)	(−0.046 − 0.023)
Constant	68.965***	58.734***	69.635***	57.790***
Random effects				
Income		0.001		0.001
LAD (constant)	2.135	1.406	2.012	1.072
Residual	553.431	551.187	551.429	548.935
Number of respondents	**21,019**	**21,019**	**22,826**	**22,826**
Number of LADs	**315**	**315**	**315**	**315**

*Source*: UKHLS Wave 5. Confidence intervals in parenthesis.

*** *p* < .001

** *p* < .01

* *p* < .05.

Fifth, we run additional analysis in order to try establish that it is exposure to inequality that drives our results. As *The English Atlas of Inequality* highlights, unequal localities also tend to be more prosperous localities: “… the most unequal locations are also the wealthiest, and many of the least unequal are in fact relatively poor from a household income point of view” (Rae & Nyanzu, 2019a, p. 30). This is reflected in our LAD‐level data (Figures A7), where the Gini measure of income inequality and median household income correlate at 0.56.

To try and ascertain that it is exposure to inequality that really drives our results, we run analysis in which we interact individual household income with LAD median household income, rather than with the Gini coefficient. We report results of the two interactions side‐by‐side in Table A8 to facilitate comparison between the two and illustrate results of the LAD median income interaction in Figures A8 and A9. On the basis that there is an interaction effect for the LAD Gini coefficient but not LAD median income, we conclude that it is exposure to contrasts that plays the critical role in the paradox of local inequality.

**TABLE A8:** Probing the effects of LAD inequality versus income by household income

Fixed effects	(1) LAD Gini	(2) LAD median income
LAD‐level		
LAD population (000s)	0.001	0.001
	(−0.001 − 0.004)	(−0.001 − 0.004)
LAD % BAME	−0.030	−0.029
	(−0.071 − 0.011)	(−0.070 − 0.013)
LAD median income (£000s)	−0.182*	−0.056
	(−0.382 − 0.019)	(−0.324 − 0.211)
LAD inequality (Gini)	45.967***	14.103
	(18.273 − 73.661)	(−7.365 − 35.571)
	
Cross‐level interaction		
LAD variable * income	−0.943***	−0.004
	(−1.472 − −0.414)	(−0.008 − 0.001)
Individual‐level		
Net household income (£000s)	0.379***	0.142**
	(0.195 − 0.563)	(0.027 − 0.257)
Male	−0.505	−0.493
	(−1.207 − 0.197)	(−1.195 − 0.209)
Age	−0.206***	−0.205***
	(−0.314 − −0.098)	(−0.313 − −0.097)
Age^2^	0.003***	0.003***
	(0.002 − 0.004)	(0.001 − 0.004)
BAME	3.071***	3.073***
	(1.995 − 4.146)	(2.000 − 4.146)
Graduate	2.364***	2.397***
	(1.654 − 3.073)	(1.681 − 3.113)
Unemployed	−4.326***	−4.393***
	(−6.187 − −2.465)	(−6.258 − −2.527)
Belongs to a religion	1.839***	1.846***
	(1.082 − 2.596)	(1.088 − 2.604)
Political leanings	2.395***	2.395***
	(1.581 − 3.210)	(1.579 − 3.212)
	−0.537	−0.565
	(−1.455 − 0.381)	(−1.482 − 0.353)
Lives in the same LAD	−1.719***	−1.705***
	(−2.789 − −0.650)	(−2.774 − −0.635)
	
Constant	60.020***	67.904***
Random effects		
Income	0.001	0.001
LAD (constant)	0.751	0.537
Residual	552.264	552.649
Number of respondents	**24,943**	**24,943**
Number of LADs	**315**	**315**

*Source*: UKHLS Wave 5. Confidence intervals in parenthesis.

*** *p* < .001

** *p* < .01

* *p* < .05.
